# Identification of a Unique Genomic Region in Sweet Chestnut (*Castanea sativa* Mill.) That Controls Resistance to Asian Chestnut Gall Wasp *Dryocosmus kuriphilus* Yasumatsu

**DOI:** 10.3390/plants13101355

**Published:** 2024-05-14

**Authors:** Muriel Gaudet, Paola Pollegioni, Marco Ciolfi, Claudia Mattioni, Marcello Cherubini, Isacco Beritognolo

**Affiliations:** CNR Istituto di Ricerca Sugli Ecosistemi Terrestri IRET, Via Guglielmo Marconi, 2, 05010 Porano, TR, Italy; paola.pollegioni@cnr.it (P.P.); marco.ciolfi@cnr.it (M.C.); claudia.mattioni@cnr.it (C.M.); marcello.cherubini@cnr.it (M.C.)

**Keywords:** Asian chestnut gall wasp (ACGW), sweet chestnut, resistance, metabolic gene cluster (MCG), pool-seq, GWAS, genomics, *Dryocosmus kuriphilus*, *Castanea sativa*

## Abstract

The Asian chestnut gall wasp (ACGW) (Hymenoptera *Dryocosmus kuriphilus* Yasumatsu) is a severe pest of sweet chestnut (*Castanea sativa* Mill.) with a strong impact on growth and nut production. A comparative field trial in Central Italy, including provenances from Spain, Italy, and Greece, was screened for ACGW infestation over consecutive years. The Greek provenance Hortiatis expressed a high proportion of immune plants and was used to perform a genome-wide association study based on DNA pool sequencing (Pool-GWAS) by comparing two DNA pools from 25 susceptible and 25 resistant plants. DNA pools were sequenced with 50X coverage depth. Sequence reads were aligned to a *C. mollissima* reference genome and the pools were compared to identify SNPs associated with resistance. Twenty-one significant SNPs were identified and highlighted a small genomic region on pseudochromosome 3 (Chr 3), containing 12 candidate genes of three gene families: *Cytochrome P450*, *UDP-glycosyltransferase*, and *Rac-like GTP-binding protein*. Functional analyses revealed a putative metabolic gene cluster related to saccharide biosynthesis in the genomic regions associated with resistance that could be involved in the production of a toxic metabolite against parasites. The comparison with previous genetic studies confirmed the involvement of Chr 3 in the control of resistance to ACGW.

## 1. Introduction

*Castanea sativa* Mill. (sweet chestnut) is one of the few tree species that can be found as natural stands and cultivated orchards throughout the Mediterranean region, from the Atlantic coast of the Iberian Peninsula to the Caspian Sea, from sea level up to 1500 m in Spain and Sicily [1]. *C. sativa* is also one of the tree species most associated with human activities [2]. It is a multipurpose tree species of high ecological and economic importance. In particular, sweet chestnut cultivation plays a key role in the economic development of the mountainous areas in Italy, Greece and North-Western Spain [3,4,5]. These three countries belong to the most important producers of chestnut fruits in Europe, together with France and Portugal [6].

In the last three centuries, *C. sativa* has had to face the attacks of three main pests: ink disease (*Phytophtora* spp.) in the 19th century, chestnut blight (*Chryphonectria parasitica* (Murrill) M.E. Barr) in the middle of the 20th century, and Asian chestnut gall wasp (ACGW), *Dryocosmus kuriphilus* Yasumatsu, at the beginning of the 21st century [4]. Native to eastern Asia, the ACGW is a parthenogenetic and univoltine gall maker Hymenoptera that infests all species and hybrids of the genus *Castanea* [7]. ACGW was first introduced into the Piemonte region in 2002 through propagation material of hybrid chestnut [8]. It rapidly spread across Italy within ten years, becoming the most severe insect pest of chestnut and reducing the chestnut nut yield up to 80% [9].

Parthenogenic females lay eggs inside buds during the summer and early instar larvae overwinter inside buds. In spring, at bud burst, they induce the formation of galls composed of plant tissues. Larvae feed inside these galls and adult wasps emerge in summer. Galls, induced by insect-derived stimuli, disrupt the twig growth and reduce fruiting [10]. Currently, there are no specific and effective chemical products for the control of this parasite. In the *C. sativa* distribution range, there are no natural enemies capable of regulating the pest populations. Therefore, the only way to fight against ACGW relies on biological control by the introduction of its exotic parasitoid, the Hymenoptera *Torimus sinensis* Kamijo.

Variability in the intensity of ACGW attacks, up to resistance, was observed in the different species of chestnut [11,12,13]. Dini et al. [14] and Lombardero et al. [13] highlighted the defensive patterns of chestnut against ACGW and identified the hypersensitive reaction (HR) as a mechanism of resistance in resistant hybrid genotypes. Although transcriptome analyses, performed mainly on *Castanea mollissima* (*C. mollissima*) [10,15,16,17], lead to the identification of different groups of candidate genes, a definite and accurate understanding of the genetic basis of the resistance to ACGW is still missing.

Recently, genomic approaches such as “genome-wide association studies (GWASs)” allow trait-associated variants (TAVs) to be identified thanks to the representation of all population genetic variation randomized by multiple generation recombination events [18]. However, the whole-genome resequencing of a high number of individuals at relatively high sequencing depth, as required for GWAS, is still expensive. For this, an alternative method has been developed based on bulk segregant analysis (BSA) and NGS technologies: the pool-seq, also named pool-GWAS or extreme-phenotype GWAS (XP-GWAS) [18,19,20]. The efficiency of this approach mainly depends on the pool size and on the depth of genome coverage, but also on the availability of a high-quality reference genome for the studied species. Pool-seq was successfully performed in many studies, such as in *Drosophila melanogaster* [21], *Arabidopsis halleri* [22], *Coffea arabica* [23], and *Eruca sativa* [24].

The genetics and molecular mechanisms of resistance to ACGW have been investigated in the Asian *Castanea* species and interspecific hybrids, but this topic is largely unexplored in *C. sativa*, despite its high susceptibility. Therefore, the objective of this study was to investigate the genetic basis of the resistance of *C. sativa* to ACGW. We evaluated the severity of ACGW attacks in a common garden comparative field trial on adult trees originated from European natural populations. In the Greek provenances, we observed a high number of gall-free plants, together with normally infested ones. This contrasting plant material was suitable for a GWAS approach. For this, we carried out a pool-seq experiment to identify the SNPs and candidate genes (CGs) associated with ACGW resistance. A single small genomic region on chromosome 3 was tightly associated with the resistant phenotype, and bioinformatics analyses identified a putative metabolic gene cluster. This is the first report about mechanisms of *C. sativa* resistance to ACGW and opens new perspectives for further studies and breeding programmes.

## 2. Results

### 2.1. ACGW Infestation Variation, Genotype Selection, and Pool Sequencing

**Figure 1:**
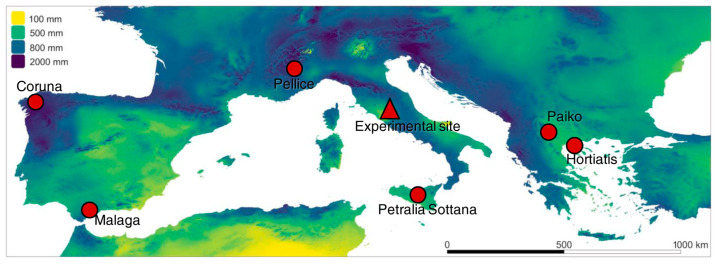
Location of *C. sativa* provenances and the experimental site with their precipitation mean. Climatic data from WorldClim v2 [25].

**Figure 2:**
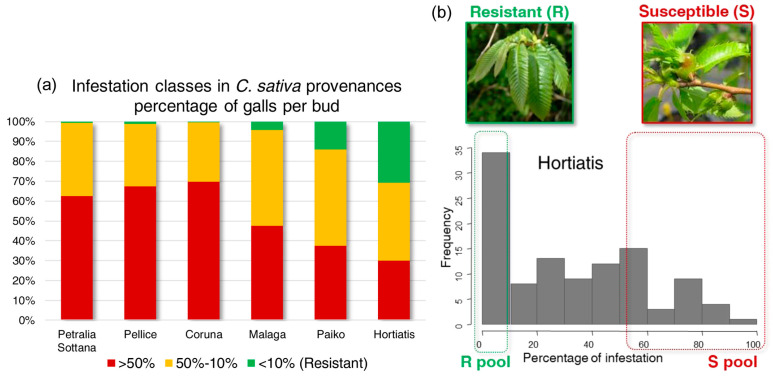
Infestation of plants. Percentage of galls per bud for all plants of each *C. sativa* provenance, year 2015. (**a**) Plants were classified in three categories according to the percentage of galls per bud: greater than 50% (red), between 50 and 10% (yellow) and less than 10% (green). Plants in this ultimate category were considered as resistant. (**b**) Distribution of the percentage of infestation in the Greek provenance Hortiatis. Dotted green rectangle indicates the portion of plants selected for the constitution of the resistant DNA pool (R pool) and the dotted red rectangle indicates those for the susceptible DNA pool (S pool). In photos are an example of a resistant plant (on the **left**) and a susceptible plant (on the **right**).

The Greek provenances showed a higher occurrence of resistant plants (24%) than the Italian and Spanish provenances (1.8% and 0.9%, respectively) (Figure 1 and Figure 2).

Hortiatis provenance had the highest fraction of gall-free plants (31%, Figure 2a). The normality of bud infestation distribution was tested on each provenance, and Coruna, Malaga, Sicilia, and Paiko fitted the normal distribution. Pellice showed a slightly skewed plot whereas Hortiatis showed a highly skewed distribution with a peak of observations in the lower tail of data distribution (Figure 2b). Sequencing of the two DNA pools resulted in more than 394 × 10^6^ and 432 × 10^6^ raw reads in the bulk of resistant (R) and susceptible (S) plants, respectively.

### 2.2. Identification of Single Nucleotide Polymorphisms (SNPs) Associated with ACGW Resistance

**Figure 3:**
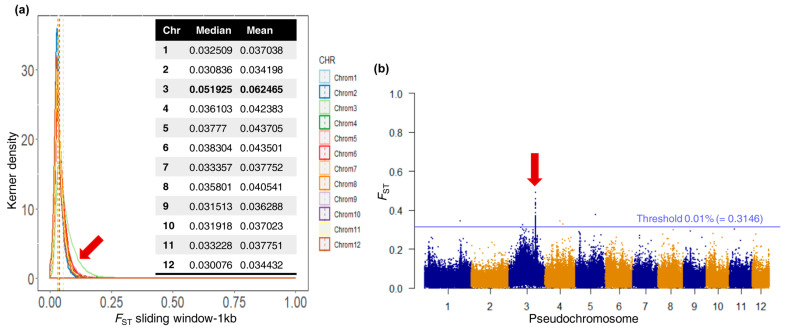
Genetic differentiation index (F_ST_) calculated on 1 kb windows. (**a**) The F_ST_ median and mean of 1 kb windows were calculated for each pseudochromosome. The red arrow highlights pseudochromosome 3. (**b**) Manhattan plot of the 1 kb window F_ST_ along each pseudochromosome. The red arrow highlights pseudochromosome 3.

After trimming, filtering, aligning to the *C. mollissima* reference genome, and filtering for low mapping quality, we obtained a total of 314 × 10^6^ and 315 × 10^6^ reads for the R and S pools, respectively. This allowed for the identification of a total of 13,701,957 SNPs. The kernel density plots of the genetic differentiation index (F_ST_) distributions calculated over 1 kb sliding windows for each Chr highlighted a higher genomic differentiation (between S and R pool) in Chr 3 than in the remaining eleven Chrs (Figure 3a). The overall genetic differentiation was calculated through the median F_ST_ of the 275,709 1 kb windows and was equal to 0.035. All the median F_ST_ values per Chr were under 0.04 (0.030 up to 0.038), except for Chr 3, which displayed a median F_ST_ equal to 0.052 (Figure 3a). Finally, the 1 kb window F_ST_ values were displayed along each Chr as Manhattan plots (Figure 3b) and confirmed that Chr 3 was a candidate for deeper analysis.

**Figure 4:**
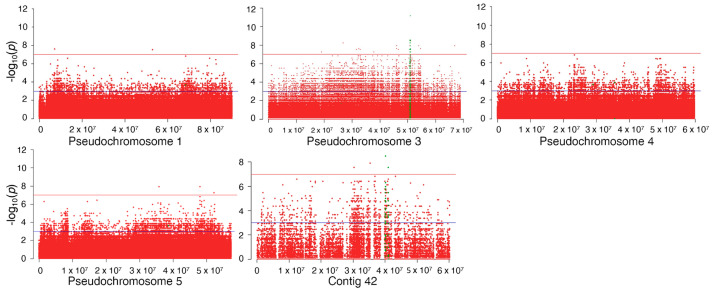
Manhattan plots of –log_10_ *p*-values of Fisher exact test at individual SNP level for the five contigs with 1 kb F_ST_ windows over the significance threshold of 0.01%. SNPs within these significant windows are represented in green and those with a –log_10_ *p*-value upper than the threshold of 5 × 10^−8^ (red line) were considered associated with Asian chestnut gall wasp resistance.

Since the F_ST_ analysis was performed on a pool of genotypes, an extremely stringent threshold of 0.01% of the F_ST_ distribution upper tail was chosen to avoid false-positive association. Thirty-seven 1 kb windows had F_ST_ over the threshold, spanning five contigs and corresponding to 774 SNPs (Figure 4). Among these 37 1 kb windows, 31 belonged to Chr 3. To confirm the significant difference in allele frequency between the R and S pools, a Fisher exact test was carried out on each individual SNP in the 1 kb windows of interest, and SNPs with a *p*-value lower than the threshold of 5 × 10^−8^ were considered associated with ACGW resistance (Figure 4).

**Figure 5:**
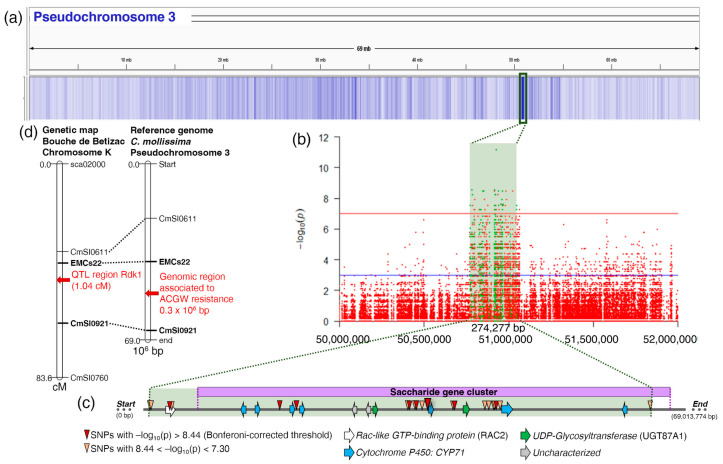
Genomic region associated with Asian chestnut gall wasp resistance. (**a**) Heatmap of Fisher exact test *p*-values at individual SNP level along pseudochromosome 3. A green rectangle highlights the genomic region with the highest *p*-value. (**b**) Zoom-in the region of interest with a Manhattan plot of Fisher exact test. The red line indicates the 5 × 10^−8^ significance threshold. SNPs within significant 1 kb windows at F_ST_ level are in green. A green rectangle highlights the genomic region with the significant SNPs. (**c**) Zoom-in the genomic region with gene annotation. Significant SNPs are indicated by a triangle with colour scale representing *p*-value. A purple rectangle indicates the saccharide metabolic gene cluster. (**d**) Genetic map of “Bouche de Betizac” Chromosome K, with QTL region Rdk1 identified by Torello et al. [26] compared to the *C. mollissima* reference genome with the genomic region identified in this study. The length of the chromosome bar is proportional to the map distance in centimorgan for “Bouche de Betizac” and to the sequence length for *C. mollissima*. Bridge SSR markers are linked with dotted lines, and the closest SSR markers (EMCs22 and CmSI0921) delimiting the region of interest are in bold.

With these criteria, 21 SNPs on Chr 3 and two SNPs on contig 42 (globally spanning 274,277 bp) were identified as being associated with ACGW resistance. Seven of these SNPs were significant after Bonferroni correction (Figure 5c). The subsequent analysis was focused on Chr 3, since the two SNPs on contig 42 were isolated polymorphisms not mapped to a Chr (Figure 5).

The projection of the boundary SSR markers nearest to Rdk1 QTL for ACGW resistance [26] onto the *C. mollissima* reference genome allowed us to compare the position of this QTL with the genomic region identified in our study (Figure 5d). The two regions were on the same pseudochromosome, Chr 3, and our genomic region falls within the interval of the QTL flanking markers EMCs22 and CmSI0921.

### 2.3. Analysis of Genomic Regions Associated with ACGW Resistance

The genomic region identified on Chr 3 contained 12 gene models for which the functional annotation was obtained with Blast2GO (Supporting tables: Table S1). Among them, *seven premnaspirodiene oxygenase-like* genes belonging to the Cytochrome P450 (CYP; P450) family, two *UDP-glycosyltransferase 87A1-likes*, and an *Rac-like GTP-binding protein*, *Rac2*, were identified. Two gene models remained uncharacterized (Figure 5c). Only two significant SNPs were within the coding sequence of the gene *Rac2*, whereas the others were in intergenic position. Assuming that the promoters were located 2 kbp upstream the transcription start site [27], two SNPs were within the expected promoters of two *P450* genes. The SNP with the most significant *p*-value in the Fisher exact test was found in the promoter of the *P450* gene, together with another SNP.

**Figure 6:**
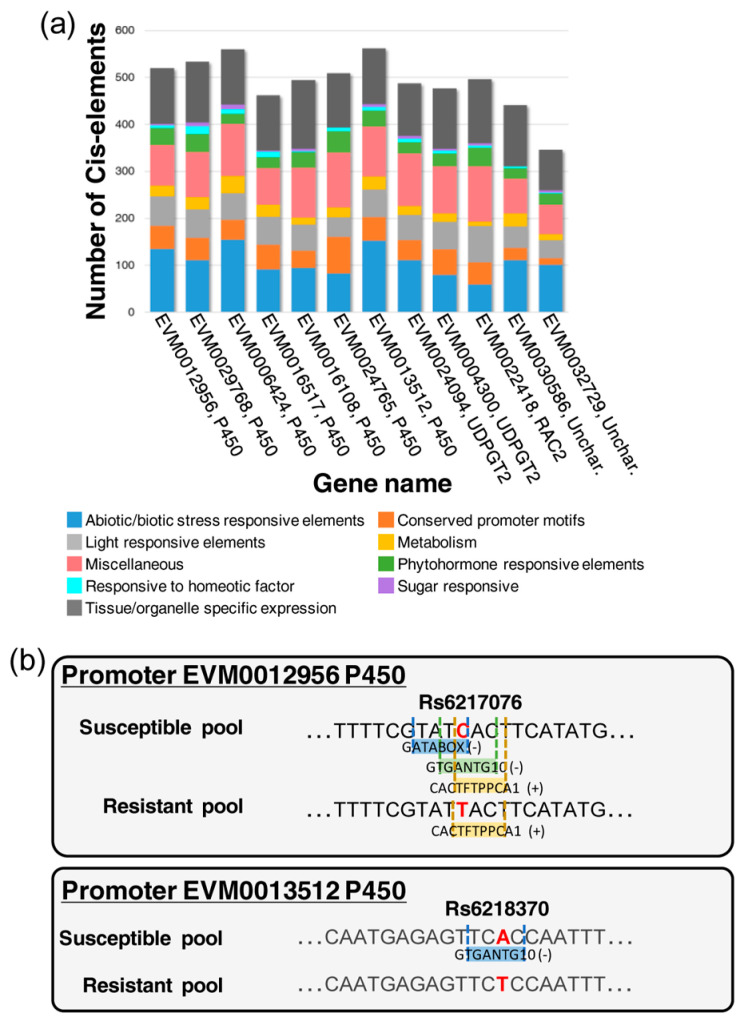
Putative cis-acting regulatory elements (CREs) found in gene promoters. (**a**) Putative CREs of the 12 candidate genes were categorized and represented by colour. The bar height is proportional to the number of cis-elements in each category. (**b**) For each SNP present in promoter regions, CRE position and allelic variant are represented for the susceptible and resistant pools. The name of the SNP is indicated over the sequence. The name of the CRE is indicated in its correspondingly coloured box; the cis-element orientation is indicated by (+) for sense and (-) for anti-sense.

The promoters of the 12 candidate genes were analyzed to predict cis-acting regulatory elements (CREs) (Supporting data: Data S1). A total of 199 different CREs were found on both sense and anti-sense strands. They were classified in the following nine main groups based on their putative function: abiotic/biotic stress responsive elements, phytohormone responsive elements, light responsive elements, responsive to homeotic factors, metabolism, sugar responsive, tissue/organelle specific expression, conserved promoter motifs, miscellaneous (which includes all CREs with functions not falling into the eight categories or not yet defined); a CRE may belong to different groups. All categories were present in all the gene promoters analyzed and the three most represented categories were tissue/organelle-specific expression, abiotic/biotic stress, and miscellaneous (Figure 6a, Supporting data: Data S1). Many CREs were involved in drought, dehydration, cold, salinity, pathogen stresses, and disease responses, and some of them were also phytohormone-responsive (W-box, WRKY). We found a high frequency of cytokinin responsiveness (ARR1AT), hormone-responsive CREs associated with ABA (ABRE), auxin (ARF, AuxRE), gibberellins (GARE-motifs), and ethylene (ERE) (Supporting data: Data S1). These identified CREs indicate that the genes may be regulated by various environmental stresses and phytohormones. The sequence variation of SNPs associated with ACGW resistance within promoters resulted in a loss of CREs (Figure 6b).

Finally, the whole sequence of Chr 3 was analyzed to look for metabolic gene clusters (MGCs) using plantiSMASH [28] and two MGCs were found: one MGC for terpenes (located from 2580.58 to 2668.97 kb (88.39 kb), with a core domain composed of terpene synthase, transferase, and cytochrome P450) and a second MGC for saccharides from 50,800.14 to 51,059.47 kb (259.33 kb) with a core domain composed of glycosyltransferase UDPGT_2 and cytochrome P450 (Supporting data: Figure S1). The saccharide MGC corresponds exactly to the genome region associated with ACGW resistance identified by this study, and only the candidate gene *Rac2* was outside this MGC (Figure 5c).

## 3. Discussion

### 3.1. ACGW Inheritance and Pool-Seq Experiment

High genetic variability in response to ACGW attacks, up to resistance, was observed in our experimental field of *C. sativa* [11,12]. Resistance to ACGW was first found in Asian species of *Castanea* (*C. mollissima* and *C. crenata*) and then in some *C. sativa* cultivars [13,29]. However, to date, resistance to ACGW has been poorly documented in natural populations of *C. sativa*. The high number of resistant plants observed in the Greek provenances, especially in Hortiatis, give us a great opportunity to study the genetic basis of the natural resistance to ACGW in *C. sativa*. In the “*Bouche de Bétizac*” hybrid cultivar (*C. sativa* × *C. crenata*), Torello Marinoni et al. [30] found a simple Mendelian inheritance of ACGW resistance, which was confirmed by the mapping of a single major QTL of the resistance trait [26]. The observed distribution of ACGW infestation in Hortiatis population suggests an oligogenic or monogenic inheritance that could explain the excess of gall-free plants in the tail of data distribution. These conditions allowed us to design a pool-seq GWAS experiment based on BSA to test hypotheses on the inheritance model.

As indicated by Schlötterer et al. [20] and Yang et al. [18], the power of the pool-seq strategy is affected by the precision of phenotyping, the pool size, and the depth of sequencing, all parameters that we considered in our research. We were able to pool a total of 25 individuals per group (resistant and susceptible plants), a number which can be considered the lower threshold for sample size in these types of experiments [20]. Nevertheless, different studies demonstrated the validity of the method with a similar, or even smaller, pool size [22,23,24,31]. In our study, the selection of resistant and susceptible individuals was based on accurate screening of phenotypes performed over multiple years. This approach, together with a relatively high sequencing depth (average: 50–60X), the availability of a contiguous and complete genome for *C. mollissima* (99.75% of the sequences anchored onto pseudochromosomes; [32]), the application of rigorous bioinformatic pipelines, and a stringent threshold for identifying statistically significant SNPs, allowed us to obtain clear and sound results.

### 3.2. A Single Genomic Region Involved in ACGW Resistance

The median F_ST_ calculated on all the 1 kb windows generated for the entire genome indicated an overall genome similarity between the two pools and the absence of population structure, which were required conditions for the identification of causative SNPs. Stringent statistical thresholds were used to avoid false-positive associations. Analysis at the chromosome level highlighted a single, relatively narrow, region of 274.3 × 10^−3^ bp in Chr 3, where 21 significant SNPs were found. This result agreed with our initial hypothesis and with the work of Torello Marinoni et al. [26], who found a major QTL for resistance to ACGW on Chr 3 of the hybrid “*Bouche de Bétizac*”. Thanks to the SSR bridge markers (EMCs22 and CmSI0921), we compared the region identified in our study with the one from Torello Marinoni et al. [26] and found that they were on the same Chr in the interval delimited by the two bridge SSRs. Unfortunately, although the Rdk1 QTL interval was narrow, no close markers were available to project it on the genome sequence with sufficient precision. Since the genome interval defined by the flanking SSRs was large, the possibility of different genome regions targeted by these two studies cannot be ruled out. Although both studies highlight the involvement of Chr 3, different regions and genes of this Chr could control ACGW resistance in *C. crenata* and *C. sativa*, respectively. For example, a similar situation is known for sex traits in dioecious *Populus* species, with closely related species that evolved different sex-determining regions on the same chromosome [33].

### 3.3. Candidate Genes

The twenty-one SNPs associated with ACGW resistance were distributed in a genomic region containing 12 gene models, corresponding to only three gene families. The *rac-like GTP-binding protein Rac2* was present in a single copy. Rac, also named Rop, are small GTPases belonging to the Rho family, which plays important roles in various cellular processes from cellular functions, growth, development, and abiotic stress signalling to plant defence [34,35,36]. In *Arabidopsis*, *Rac2/Rop7* was found to play a role in xylogenesis [37], while in tomato, it was found to be involved in plant immune response to pathogen infection [38]. *Rac2* is an interesting candidate gene, as the Rac/Rop family plays a crucial role in plant immunity [36].

Seven genes of premnaspirodiene oxygenase-like family were present in the genome region associated with ACGW resistance. These genes belong to the large family of Cytochrome P450s (CYPs), which is characterized by a wide diversity of functions, being responsible for the catalyzation of more than 20 types of reactions with a key role in the diversification and functional modification of plant metabolism [39,40]. They mainly participate in hormone signalling for the regulation of plant responses under stress conditions [39,41]. Premnaspirodiene oxygenase is part of the largest CYP71 clan and interacts with sesquiterpenes, resulting in a solavetivone (a potent antifungal phytoalexin [42]) or a regulator of downstream defence-related genes [41]. CYP71 is also involved in the biosynthesis of a cyanogenic glucoside, which is highly toxic to most living organisms and protects plants from herbivores [43,44].

The third gene family identified in this study is UDP-glycosyltransferase (UGT). This enzyme glycosylates many small molecules, such as phytohormones, endogenous secondary metabolites, and xenobiotics. UGTs are involved in the responses to biotic and abiotic stresses [45,46]. More specifically, the two *C. sativa* genes associated with ACGW resistance were attributed to the UGT87 sub-family, which was found to be involved in the response to pathogens and to oxidative stress in *Arabidopsis* and *Brassica* [47].

### 3.4. Cis-Regulation

Nineteen SNPs out of the twenty-one associated with ACGW resistance were in non-coding sequences, and therefore, they could be part of CREs that regulate gene expression. Forward genetics is widely applied to detect non-coding regions, putative cis-regulatory modules (CRMs) associated with phenotypic variation [27]. In fact, a recent GWAS for plant resistance to biotic and abiotic stress has shown that more than 70% of significant functional hits are localized in intergenic regions [48]. In plants, CREs could be located kilobases to megabases away from their target genes. However, promoters are defined as being one or two kbp upstream of the transcription start site [48]. In this study, the analysis of the putative promoter region of candidate genes (2000 bp upstream ATG) revealed the presence of several CREs involved in the response to different environmental stresses. Polymorphisms in regulatory regions can change the phenotype from susceptible to resistant with, for example, the loss of binding sites for pathogen effector proteins [48]. Some of the CREs identified in *C. sativa* could be involved in the response to ACGW attack. Specifically, two SNPs associated with ACGW resistance causes the loss or change of some CREs, including the most significant SNP Rs6217076; thus, their polymorphism could have a functional role in switching the activation of downstream genes.

### 3.5. Metabolic Gene Cluster

The cluster of genes and the intergenic positions of the main SNPs associated with ACGW resistance suggested that this region could be regulated as a single block. Indeed, the search for metabolic gene clusters (MGCs) on Chr 3 identified a putative saccharide MGC exactly in the region of interest. Plant MGCs are defined as a cluster of non-homologous genes that act sequentially and together in a shared biosynthetic pathway to form a specific metabolite [49,50]. MGC genes are recruited from primary metabolism through gene duplication followed by neofunctionalization. MGCs are one of the ways that plants rapidly modify and form new chemistries in response to biotic and abiotic stresses and environmental changes [51]. Almost all plant MGCs identified to date produce defence metabolites that protect plants against parasites [49]. Of these metabolites, more than a half are associated with terpenes. The other MGC metabolites, identified up to now, are cyanogenic glycosides (N-containing compounds), alkaloids, benzenoids, phenylpropanoids, and fatty acids [50,52]. Consequently, the genomic region identified in our study is likely involved in the biosynthesis of a secondary metabolite potentially toxic to ACGW in *C. sativa*. Lombardo et al. [13] found that, contrary to the expectation, resistance of *C. sativa* to ACGW does not depend on phenols, terpenes, and primary nutrition, which is in agreement with the putative MGC associated with saccharides identified in our study. Interestingly, cyanogenic glucoside biosynthesis requires two cytochrome P450 enzymes and a UDP-glucosyltransferase, which are the genes identified in the MGC of our genomic region of interest. Indeed, MGCs for cyanogenic glucoside biosynthesis found in *Lotus japonicus*, *Sorgum bicolor,* and *Manihot esculenta* contain *CYP* genes belonging to the clan CYP71 and *UDP-glucosyltransferase UGT85* genes [53], while in *Eucalyptus*, a cyanogenic glucoside MGC contains a novel *UDP-glucosyltransferase* of class UGT87, similar to the *C. sativa* UGT identified in our study [54]. We hypothesize that the putative MGC of our study is involved in the production of a metabolite needing glycosylation, maybe in the biosynthesis of a cyanogenic glucoside.

### 3.6. Conclusions

This study provides the first genomic insight into the resistance to ACGW of natural populations of *C. sativa*. A small genomic region on Chr 3 was found to be associated with the high resistance observed in a Greek provenance. Our results confirm the involvement of Chr 3 in the vertical resistance to ACGW, as already reported for Asian *Castanea* species. The genetic basis of ACGW resistance could be specific to the Greek provenance Hortiatis and needs to be tested genetically and functionally in unrelated plant material of *C. sativa*.

The phenotypic data and the presence of an MCG suggest that the narrow genomic region of interest controls the inheritance of resistance and acts as a functional block to produce defence metabolites. The candidate genes and MGC identified lead to the hypothesis that cyanogenic compounds could be responsible for the immunity found in the Greek provenance. New transcriptome studies could clarify the transcriptional regulation of the identified MGC. The results obtained open new research perspectives to better understand the interaction between *C. sativa* and ACGW; moreover, our study provides important indications to improve plant breeding programmes and parasite control.

The resistance mechanisms found in the Greek provenance could be the result of some biotic or abiotic selective pressures other than ACGW. Given the different coevolution history with ACGW of *C. sativa* and the Asian chestnut species, it is not necessarily expected that the same defence mechanisms evolved in the different species. The data available do not allow for a comparison of the respective genomic regions involved in ACGW resistance. However, the presence of an MGC in *C. sativa* opens hypotheses on plant adaptive strategies to protect against parasites by the neofunctionalization of genes. Therefore, Chr 3 could harbour interesting gene families for the response to stress, which could have evolved differently in *C. mollissima*, *C. crenata,* and *C. sativa* through the formation of MGCs.

This study provides new knowledge on the putative mechanisms of *C. sativa* resistance to ACGW controlled by a specific MGC in a small genomic region and therefore opens perspectives for breeding programmes using new technologies, such as CRISPR/Cas9 gene editing, available in *C. sativa* [55].

## 4. Materials and Methods

### 4.1. Plant Materials

The plants analyzed were grown in a common garden comparative field trial at CNR IRET (Porano, Central Italy; Figure 1), with half-sib families originating from six natural *C. sativa* provenances from Italy (Pellice and Petralia Sottana), Spain (Coruna and Malaga), and Greece (Paiko and Hortiatis) (Figure 1). The experimental field was set up in 2002 for the European project CASCADE (EVK2-99-00065). Two natural populations from contrasting climatic conditions (mesic vs. xeric conditions) were chosen in each country and seeds of individual mother trees were collected in 2001. Seeds were germinated and grown in a nursery, and seedlings were planted according to a completely randomized design in spring 2002; for a more detailed description, see Pliura and Eriksson [56]. Today, there are about 1000 plants left in the field, which were colonized by ACGW in 2008–2009.

### 4.2. ACGW Infestation Scoring

The level of ACGW infestation was evaluated in 2014, 2015, and 2016 as described by Contarini et al. [12]. The percentage of galls per bud was calculated for all the plants of the common garden comparative field, and plants with a percentage lower than 10% were considered resistant to ACGW (Figure 2a). The data scored in 2015 were used to perform a Shapiro–Wilk test for normality on the frequency distribution of the gall infestation percentage of each provenance. A significant variation in susceptibility to ACGW among provenances and the highest resistance of Hortiatis provenance were confirmed in the different years by two independent studies [11,12]. Therefore, the plants of Hortiatis provenance were considered as a suitable model for a BSA based on a pool-seq GWAS experiment and NGS technology. The observed level of ACGW infestation was used to select two groups of plants with extreme phenotypes. Twenty-five resistant plants (infestation rate < 10%) and 25 susceptible plants (infestation rate between 52% and 91%) were selected to form two DNA pools. The pool of resistant plants was made up of 19 immune trees (gall-free) and six trees with an infestation rate between 4% and 9%. The experimental design was controlled in terms of number of half-sibs per mother to avoid an over-representation of a family, and plants were selected only within the provenance of Hortiatis to reduce the risk of spurious allelic association due to genetic divergence between provenances.

### 4.3. DNA Extraction, Quality, and Pooling

Genomic DNA was extracted from the young leaves of each of the 50 selected plants prior to bulking. A washing step before DNA purification was carried out as in the study by Xu et al. [57] to remove contaminants, such as organic molecules, and excessive water. For each sample, around 100 mg of frozen ground powder was washed twice with the washing solution (100 mM Tris-HCl (pH 8.0), 5 mM ethylenediaminetetraacetic acid (EDTA, pH 8.0), 0.35 M glucose, bovine serum albumin (BSA) 0.1% (*w*/*v*), 2% PVP (*w*/*v*), and β-mercaptoethanol 3% *v*/*v* (added before use)). Then, total DNA was extracted following the procedure of Doyle and Doyle [58,59] with a CTAB buffer (CTAB 2% (*w*/*v*), 100 mM Tris-HCl (pH 8.0), 20 mM EDTA (pH 8.0), 1.4 M NaCl, 2% PVP40 (*w*/*v*), and β-mercaptoethanol 3% *v*/*v* (added before used the solution)). The NaCl concentration was set to 1.4 M to further remove polysaccharides and PVP40 was added to remove polyphenols. Three purification steps with chloroform/IAA 24:1 were performed, and an RNase treatment was added after the first step.

The integrity, purity, and concentration of all genomic DNA were assessed by gel electrophoresis and spectrophotometric analysis. To minimize pipette-associated errors, the DNA was quantified in triplicate. Two equimolar DNA pools were constructed by combining 800 ng of each sample by manual pipetting.

### 4.4. Sequencing

Two DNA libraries were prepared and sequenced at IGA Technology Services (Udine, Italy). Briefly, the two DNA libraries were prepared using mechanic sonication fragmentation and ligation of specific adapters. After PCR enrichment, they were sequenced to an expected mean coverage of 50× using the Illumina HiSeq2500 (v4) platform (Ilumina Inc., San Diego, CA, USA) with paired-end, 125 bp reads. Quality control of each library was performed with the BioAnalyzer 2100 (Agilent Technologies, Santa Clara, CA, USA). Raw data (raw 125 bp paired-end reads) are available in the BioStudies database (http://www.ebi.ac.uk/biostudies, accessed on 14 March 2024) under accession number S-BSST1362.

### 4.5. Bioinformatics Pipeline and SNP Frequency Comparison

Raw reads were trimmed to remove low-quality bases and adapter sequences with default parameters of the Cutadapt v3.4 [60] and Erne-filter implemented in ERNE 2 [61] software. The most advanced *C. mollissima* Chinese genome assembly, ASM1418300v1, was used as the reference genome with 99.75% of the assembled sequences anchored onto the 12 *Castanea* spp. chromosomes [32]. Thus, clean sequences were mapped to the *C. mollissima* reference genome (ASM1418300v1) available on NCBI (https://www.ncbi.nlm.nih.gov/data-hub/genome/GCA_014183005.1/, accessed on 12 October 2021) using bwa mem (v07-10-r789, [62]) by specifying the option –k 30 to meet the specificity of pool-seq data, increasing mapping accuracy with this semi-global mapping approach. The SAM alignment files were then converted to binary BAM files, then sorted and indexed using the software package SAMtools v1.10 [63]. The tool MarkDuplicates of the software package Picard v.1.119 (http://picard.sourceforge.net, accessed on 14 October 2021) was applied to mark and remove the optical duplicates from the alignments. The alignments were further filtered based on their mapping quality. In order to maintain high mapping quality, reads with a quality score < 20, singletons, and unproper pairs were removed from the alignments using SAMtools.

### 4.6. Statistical Analysis and Identification of Trait-Associated SNPs

Single-nucleotide polymorphisms were called with SAMtools [63], and each mpileup file created was synchronized and again filtered for base quality (Q20) using the perl script mpileup2sync.pl of the POPOOLATION2 software [64]. The synchronized (sync) output file contains the information about all the SNPs detected and the respective allele frequencies. The coverage along the genome and the coverage per quantile were calculated using the SAMtools depth and R quantile functions, respectively. We detected the coverage values at 1% increments and considered 98% as maximum coverage value for each pool. To accurately estimate allele frequency and correct for potential errors from copy number variations and mis-mappings, a minimum coverage of 20 and maximum coverage of 80 were used as thresholds for SNP identification in the dataset comparison. To identify significantly differentiated SNPs between the two pools, the F_ST_ was calculated with fst-sliding.pl in POPOOLATION2, using a sliding-window approach with a window size of 1 kb and a step size of 1 kb. The POPOOLATION2 script subsample-synchronized.pl was also used to select all the SNPs with a maximum coverage of 80 or 100 using the max-coverage option. The method parameter “without_replace” was applied to the normalization process. Finally, the F_ST_ values were displayed along each Chr as Manhattan plots using the qqman R package [65].

Genomic regions presenting significant genetic differentiation between the two pools, corresponding to an F_ST_ value falling in the upper 0.01% tail of the F_ST_ distribution range, were screened at windows and the individual SNP level, performing a Fisher exact test with the Perl script fisher-test.pl in the POPOOLATION2 software. The SNPs with a *p*-value lower than the strict threshold of 5 × 10^−8^, which is widely used as the standard in GWAS [66], were defined as significantly differentiated and associated with the trait of interest. We also calculated the Bonferroni-corrected *p*-value threshold for multiple testing at α = 5%, i.e., 3.65 × 10^−9^.

### 4.7. Functional Annotation and Comparative Mapping

Genomic annotations and sequences of *C. mollissima* genome version 2 were downloaded from the NCBI genome assembly database (https://www.ncbi.nlm.nih.gov/assembly/GCA_000763605.2/, accessed on 18 April 2023). Significant SNPs were mapped to the reference genome and checked to be part of genes or potential gene promoters. The gene models closest to the SNPs were imported into Blast2GO (version 6.0.3; [67,68,69]) to obtain functional annotation. GO (Gene Ontology) and KEGG (Kyoto Encyclopedia of Genes and Genomes) annotations were obtained using the default settings of Blast2GO. The upstream region of genes (2000 bp, from −2000 to the start site ATG) was analyzed using New Place (https://www.dna.affrc.go.jp/PLACE/?action=newplace; accessed on 15 February 2023 [70]) online tools to predict cis-acting regulatory elements (CREs). Identified CREs were classified into nine major groups according to their role in gene regulation. A search for metabolic gene clusters (MGCs) was also performed using plantiSMASH (http://plantismash.secondarymetabolites.org/; accessed on 22 June 2023 [28]) to identify secondary metabolite biosynthesis gene clusters.

To compare our data to the QTL for ACGW resistance in chestnut found by Torello Marinoni et al. [26], the position of genetic markers of the linkage group containing the QTL were searched in the *C. mollissima* reference genome. The physical maps were drawn and compared to the genetic map using MapChart 2.32 [71].

## Data Availability

Raw data (raw 125 bp paired-end reads) are available in the BioStudies database (http://www.ebi.ac.uk/biostudies; accessed on 14 March 2024) under accession number S-BSST1362.

## Supplementary Materials

The following supporting information can be downloaded at https://www.mdpi.com/article/10.3390/plants13101355/s1: Figure S1: Results of the search for metabolic gene clusters (MGCs) on pseudochromosome 3 (GWHANWH00000102) using plantiSMASH; Table S1: Annotation by Blast2GO software of the 12 gene models close to the significant SNPs associated with ACGW resistance; Data S1: Cis-acting regulatory elements (CREs) identified with New Place in the promoter (2000 bp, from −2000 to the start site ATG) of the 12 candidate genes of our study.
