# Supplementary material for: Identification of a Unique Genomic Region in Sweet Chestnut (Castanea sativa Mill.) That Controls Resistance to Asian Chestnut Gall Wasp Dryocosmus kuriphilus Yasumatsu
Source: Plants (Basel). 2024 May 14;13(10):1355. doi: 10.3390/plants13101355 (PMC11125237; doi:10.3390/plants13101355)
Supplement: Supplementary file 1 [file plants-13-01355-s001.zip › Figure S1.pdf]

Figure S1

Plant Secondary Metabolite Analysis
Version 1.0.0-beta

Select Gene Cluster:
Overview 1 2

Identified secondary metabolite clusters

Search by locus tag, Pfam ID, or biosynthetic type:

Cluster	Record	Type	From	To	Size (kb)	Core domains	CD-HIT Clusters	Most similar known cluster	MIBiG BGC-ID
Cluster 1	GWHANWH00000102	Terpene	2580576	2668971	88.39	Terpene_synth_C, Transferase, p450	3	-	-
Cluster 2	GWHANWH00000102	Saccharide	50800144	51059473	259.33	UDPGT_2, p450	4	-	-

Showing 1 to 2 of 2 entries

If you have found plantiSMASH useful, please cite us.

GWHANWH00000102 - Cluster 2 - Saccharide

Gene cluster description

GWHANWH00000102 - Gene Cluster 2. Type = saccharide. Location: 50800144 - 51059473 nt. Click on genes for more information.

Show pHMM detection rules used

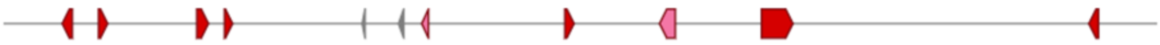

< - reset + >

Genes: hide

Locus tag	Functional annotation	From	To	Strand	Category	Domains
EVM0029768	-	50823921	50825996	-	Cytochrome 450	p450 (E=1.1e-97)
EVM0006424	-	50831360	50833216	+	Cytochrome 450	p450 (E=1e-30)
EVM0016517	-	50851432	50853814	+	Cytochrome 450	p450 (E=4.6e-99)
EVM0024765	-	50857087	50858748	+	Cytochrome 450	p450 (E=2.1e-39)
EVM0030586	-	50885224	50885817	-	Other genes	n/a
EVM0032729	-	50892656	50893720	-	Other genes	n/a
EVM0024094	-	50897407	50898763	-	Glycosyltransferase	UDPGT_2 (E=1.5e-13)
EVM0012956	-	50926639	50928530	+	Cytochrome 450	p450 (E=1.1e-85)
EVM0004300	-	50946001	50949215	-	Glycosyltransferase	UDPGT_2 (E=2.1e-13)
EVM0013512	-	50966821	50973259	+	Cytochrome 450	p450 (E=8.6e-81)
EVM0016108	-	51033725	51035696	-	Cytochrome 450	p450 (E=7.9e-97)

Show:

☑ biosynthetic genes ☑ other genes

Legend:

Cytochrome 450 | Glycosyltransferase | (Other) Biosynthetic Genes | Other Genes

biosynthetic genes | transport-related genes | regulatory genes | other genes
